# Elastin aging and lipid oxidation products in human aorta

**DOI:** 10.1016/j.redox.2014.12.008

**Published:** 2014-12-18

**Authors:** Kamelija Zarkovic, Pauline Larroque-Cardoso, Mélanie Pucelle, Robert Salvayre, Georg Waeg, Anne Nègre-Salvayre, Neven Zarkovic

**Affiliations:** a Division of Pathology, Clinical Hospital Centre, Zagreb, Croatia; b Inserm UMR-1048, Toulouse, France; c University of Toulouse, Toulouse, France; d Institute of Molecular Biosciences, University of Graz, Austria; e Rudjer Boskovic Institute, LabOs, Zagreb, Croatia; f University for Applied Sciences Baltazar, Zaprešić, Croatia

**Keywords:** **4**-HNE, 4-hydroxynonenal, ALEs, advanced lipoxidation end products, AGEs, advanced glycation End Products, ECM, extracellular matrix, MMPs, matrix metalloproteases, SMC, smooth muscle cells, 4-hydroxynonenal, Elastin, Atherosclerosis, Smooth muscle cells, Extracellular matrix, Aging

## Abstract

Vascular aging is associated with structural and functional modifications of the arteries, and by an increase in arterial wall thickening in the intima and the media, mainly resulting from structural modifications of the extracellular matrix (ECM) components. Among the factors known to accumulate with aging, advanced lipid peroxidation end products (ALEs) are a hallmark of oxidative stress-associated diseases such as atherosclerosis. Aldehydes generated from the peroxidation of polyunsaturated fatty acids (PUFA), (4-hydroxynonenal, malondialdehyde, acrolein), form adducts on cellular proteins, leading to a progressive protein dysfunction with consequences in the pathophysiology of vascular aging. The contribution of these aldehydes to ECM modification is not known. This study was carried out to investigate whether aldehyde-adducts are detected in the intima and media in human aorta, whether their level is increased in vascular aging, and whether elastin fibers are a target of aldehyde-adduct formation. Immunohistological and confocal immunofluorescence studies indicate that 4-HNE-histidine-adducts accumulate in an age-related manner in the intima, media and adventitia layers of human aortas, and are mainly expressed in smooth muscle cells. In contrast, even if the structure of elastin fiber is strongly altered in the aged vessels, our results show that elastin is not or very poorly modified by 4-HNE. These data indicate a complex role for lipid peroxidation and in particular for 4-HNE in elastin homeostasis, in the vascular wall remodeling during aging and atherosclerosis development.

## Highlights

• Elastin alteration, degradation, and loss in aged arteries.
• Increased lipid oxidation products (4-HNE) accumulation on cells and ECM in the intima and adventitia, and at a lesser extent in the media of aged patients.
• No modification of elastin by 4-HNE in human vessels.
• Abundant 4-HNE-protein adducts in aortic smooth muscle cells.

## Graphical abstract

**Figure:**
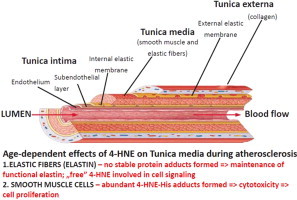

## Introduction

Aging is the largest known risk factor for most human diseases [1], [2], [3], [4]. The aging process generates a progressive loss of biological functions and abilities to manage metabolic changes, with particular impact on the development of cardiovascular diseases [5], [6], which represent the major cause of morbidity and mortality in aged people [7], [8]. It is very important to understand the physiological mechanisms involved in the natural process of aging, to develop new prevention, diagnosis and treatment approaches, allowing to slow down the onset of aging consequences.

In normal arteries, the proteins of the extracellular matrix (ECM) (collagen, elastin, fibrillin, glycoproteins and proteoglycans) produced by smooth muscle cells (SMC) ensure the stability, resilience, and compliance of arteries [9]. Collagen and elastin, two major scaffolding ECM proteins provide structural integrity and elasticity to the vessels, allowing them to stretch while retaining their ability to return to their original shape when the pressure is over. Vascular aging is most of the time associated with structural and functional modifications of the arteries, even in healthy elderly, and particularly by an increase in arterial wall thickening in the intima and the media, mainly resulting from the accumulation and structural modification of ECM components and a disorganization of SMC [10], [11]. Increased expression of matrix metalloproteases, (MMP-2, MMP-1, MMP-9), as well as the decreased expression of tissue inhibitors of MMPs (TIMPs) contribute to the fragmentation of elastic fibers [12], [13]. Increased collagen deposition and reduction of elastin content due to elastin fiber degradation, often associated with vascular calcifications, contribute to the development of arterial stiffening [5].

Arterial stiffness is characterized by structural and functional alterations of the intrinsic elastic properties of the arteries and an increased resistance to vessel deformation, resulting from a decrease in artery elasticity (compliance) and an increase in pulse wave velocity (pwv) [5], [14], generating an increased systolic pressure, with deleterious consequences on the heart, generating cardiac hypertrophy and increased ventricular oxygen consumption. Arterial stiffening is a hallmark of vascular aging, and a major risk factor for the development of cardiovascular diseases, that can be exacerbated by diabetes, hypertension or atherosclerosis. It is a direct cause of ventricular hypertrophy, renal dysfunction and stroke, independently of the other causes of vascular aging [15]. It is an independent risk factor for cardiovascular diseases, which may predispose to atherosclerosis, and *vice-versa*. The mechanisms linking these two risk factors are not known [16] .

Arterial stiffness is aggravated by the presence of advanced glycation-end products (AGEs), formed during glucose oxidation, which slowly accumulate in normal aging, and are strongly increased in diabetes [17], [18]. AGEs form cross-links on ECM proteins (on collagens; but also on elastin) [19], [20], by reacting with their lysine residues, which decreases their turnover and promotes arterial stiffness and intima-media thickness [21]. These modifications are implicated in the loss of vascular elasticity.

Though electrophilic carbonyl compounds derived from lipid peroxidation play a major involvement in the modulation of life-longevity [22], and the development of oxidative stress-associated diseases such as atherosclerosis or neurodegenerative diseases [23], [24], it is not known so far whether and how these agents are involved in the onset of vascular aging. Moreover, since arterial stiffness and atherosclerosis are independent cardiovascular risk factors, and are characteristic of vascular aging, it is difficult to link atherosclerosis and oxidized lipids to arterial stiffness development [8], [16]. Lipid peroxidation products accumulate with aging, due to a reduction in antioxidant defenses and increased oxidative stress [25], [26], [27]). Aldehydes generated from the peroxidation of polyunsaturated fatty acids (PUFA), particularly 4-hydroxynonenal (4-HNE), malondialdehyde (MDA), acrolein, share several properties with AGEs, as they form adducts on cellular proteins, particularly on histidine and cystein (4-HNE) or lysine (acrolein), leading to a progressive protein dysfunction with consequences in the pathophysiology of vascular aging [24]. As a consequence, detoxification mechanisms, including the removal of electrophiles by glutathione transferase-catalyzed conjugation, are considered as major longevity assurance mechanisms [22]. It is not known whether aldehydes accumulate on ECM proteins (elastin or collagen), and contribute to elastin aging and arterial stiffness. However the formation of 4-HNE adducts on elastin has been reported in the dermis in human patients affected with actinic elastosis, suggesting that 4-HNE generated by UVA and UVB from solar radiations, is able to modify elastin in the skin [28].

The objectives of this study were to investigate whether aldehyde-adducts are detected in the intima, media and adventitia in human aorta, whether their level is increased as function of aging, and whether elastin fibers are a target of aldehyde-adduct formation. Immunohistological and confocal immunofluorescence studies indicate that 4-HNE-adducts accumulate in aged aortas, mainly on SMC. In contrast, even if the structure of elastin fiber is strongly altered in aged vessels, our results show that elastin is not or very poorly modified by 4-HNE. These data do not rule out a role for lipid peroxidation in the metabolism (degradation, lack of renewal) of elastin in the vascular wall.

## Material and methods

### Aortas recovery

The material for the study consisted of 59 human abdominal aorta specimens removed by autopsy from patients who died at the age comprised between 32 and 91 years (average 71 year-old), out of which 30 were women and 29 men. None of the deceased persons suffered from any type of connective tissue disorder, 30 were hypertensive, 31 diabetic and 17 smokers. Specimen of aorta of the 3 year-old child was taken as a healthy control sample in the study.

The autopsies were performed within 6–10 h after death in the Department of Pathology, Clinical Hospital, Zagreb. Aorta tissue was grossly inspected, cut by consecutive sections into slabs of about 5 mm thickness and fixed in 10% formalin. Upon fixation the aorta tissues were dehydrated in graded ethanol and embedded in paraffin.

### Immunohistochemistry

Formaldehyde-fixed aorta segments embedded in paraffin were cut into 10 µm sections. Monoclonal antibodies that detect 4-HNE-modified proteins were obtained from culture medium of clone “HNE g4” which was derived from a fusion of S2-Ag8 myeloma cells with B-cells of a BALBc mouse immunized with 4-HNE modified keyhole limpet hemocyanin [29]. The antibody is specific for the HNE-histidine epitope in HNE-protein conjugate. 4-HNE-lysine and 4-HNE-cysteine give 5% and 4% cross-reactivity with HNE Ig4. Immunohistochemical detection of 4-HNE adducts was done using the Envision System (Dako, Denmark), with contrast staining using hematoxylin and Masson’s trichrome. Positive reaction to HNE was stained by 3, 3-diaminobenzidine tetrachloride (DAB, Dako, Denmark), and pale red positive elastin staining with Masson trichrome, according to visually determining the relationship of immunohistochemical positive reactions with elastin [30].

Stained sections were analyzed qualitatively and semi-quantitatively on Olympus 40 light microscope. Immunohistochemical staining to 4-HNE were analyzed in the three aorta layers for each vessel specimen. The immunohistochemical 4-HNE staining was graded mainly in SMC, as follows: 0 − entirely negative, (though some SMC can be positive), 1- low positive in sporadic SMC; 2 – moderately positive in 10–30% SMC and 3-4-HNE immunopositivity present in more than 30% of SMC.

### Immunofluorescence and confocal microscopy

Aorta sections were stained with the rabbit polyclonal anti 4-HNE-Michael adduct (Calbiochem) and mouse monoclonal anti-human elastin antibodies, alexa-Fluor 488 (green)- and Alexa-Fluor 546 (red)-conjugated secondary antibody were from Molecular Probes (Invitrogen, Cergy-Pontoise, France). The slides were visualized using a Zeiss LSM 510 fluorescence confocal microscope (Le Pecq, France). Picture analysis was done using ImageJ 1.49j software.

### Atherosclerotic grade quantification

Atherosclerotic lesions in intima of aorta were analyzed on hematoxylin eosin stained slices. Intimal lesions in aorta were classified in the following subgrades:

1.Initial lesions.
1a.Fatty streak with accumulation of intracellular (macrophages and smooth muscle cells) and extracellular lipid in intima of aorta.
1b.Intimal cell mass containing smooth muscle cells and connective tissue but not lipid (“cushions”).
2.Characteristic atherosclerotic fibro-fatty plaque consisting of two morphologic components:
2a.The fibrous cap of thick fibrous connective tissue with foamy macrophages and smooth muscle cells covered by intact endothelium.
2b.Atheroma-necrotic mass of lipids that forms the middle part of the lesion with fibrous cap over it and the loss of endothelial continuity combined with infiltration of inflammatory monocytes and activated T lymphocytes.
3.Complicated atherosclerotic lesions: Thrombosis on and within the fibrous cap. Neovascularization of the cap.
4.Thinning of the underlying tunica media. Calcification within the atheroma and fibrous cap.
5.All type 3/4 complications plus ulceration of the fibrous cap.

### Statistical analyses

Estimates of statistical significance were performed by *t*-test or analysis of variance followed by multiple comparison procedure (using the SigmaStat 3.5, Systat software). Tests for normality and equal variance were performed by the Kolmogorov–Smirnov test. Differences between means values were evaluated by unpaired *t* test, to compare two groups, or by one-way ANOVA to compare group I vs the other groups, by the Holm-Sidak test. Values of *p* < 0.05 were considered significant.

## Results

### Accumulation of 4-HNE-adducts in the vessels

**Fig. 1:**
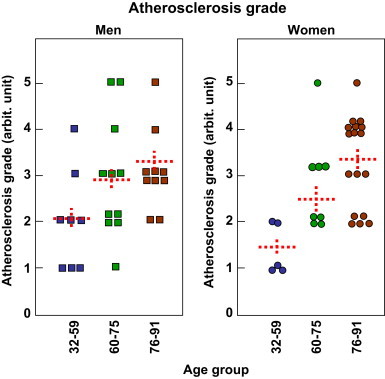
Age- and sex-related atherosclerosis grade of the different subjects. 59 subjects were analyzed, 29 men and 30 women, each divided in three groups, group I (32–59 year-old), group II (60–75 year-old), group III (76–91 year-old). The results are presented as function of the atherosclerotic grade, classified from 1 to 5 as described in the Materials and Methods section. Statistical analysis was performed using one-way ANOVA followed by Holm-Sidak test, group I being compared to group II and III (SigmaStat software). Comparison group II to group III was not significant. Mean ± SEM are indicated by the doted line. ^⁎^*p* < 0.05.

We first checked whether 4-HNE adducts accumulate in an age-dependent manner in human aortas. Three groups were defined, group I, aged 32–59 years, group II, aged 60–75 years, group III, aged 76–91 years. The characteristics of each group are described in the Materials and Methods section. The atherosclerosis grade was defined for each group, between men and women, and though the study was carried out on a small number of subjects, the data reported in Fig. 1, confirm the prevalence of the age-related atherosclerotic lesion development, and the increased atherosclerotic grade in men *vs* women, significant at least for the group I *vs* group II or *vs* group III. Noteworthy, hypertensive patients had high grade lesions, i.e. complicated atherosclerotic lesions, while in normotensive patients, atherosclerosis was mostly represented by characteristic atherosclerotic lesions, i.e. fibro-fatty plaques. These data are not shown, because most hypertensive patients were more than 65 year-old, and the intensity of the media staining by 4-HNE adducts was not significantly different from that of normotensive subjects.

Intimal thickening reflected the degree of atherosclerosis in all samples, while thickness of the tunica media did not correlate with the degree of atherosclerosis, age or even hypertension, indicating primarily constitutional differences among people.

**Fig. 2:**
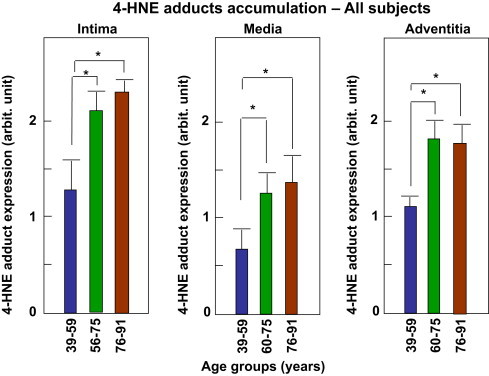
Accumulation of HNE-adducts in human aortas. Aortic sections were labeled with an anti-histidine adduct antibody and revealed by a peroxidase-conjugated secondary antibody, as described in the Material and Methods section. The intensity of HNE reaction was semi-quantitatively evaluated in SMC, as 0 (negative), 1 (low, less than 10% of 4-HNE-positive SMC), 2 (moderated, 10–30%), 3 (more than 30%). The results are presented as mean of HNE staining intensity in all 59 subjects (men + women) divided in the three groups as defined in Fig. 1: Age group I: 32–60 year-old, 13 subjects; Age group II: 60–75 year-old, 19 subjects; Age-group III: 75–91 year-old, 27 subjects. The three aortic layers (intima, media and adventitia), were analyzed for each subject. Statistical analysis was performed using one-way ANOVA followed by Holm-Sidak test, group I being compared to group II and III (SigmaStat software). Comparison group II to group III was not significant. Mean ± SEM are indicated by the doted line. ^⁎^*p* < 0.05.

The expression of 4-HNE-adducts was semi-quantitatively evaluated on all subjects and in each aortic layer, as shown in Fig. 2. Our results indicate that the expression of 4-HNE adducts is age-dependent, in each aortic layer. The differences are very strong in the intima, due to the marked development of atherosclerotic lesions in very aged people (the most important group in this study). As previously reported [23], [24], 4-HNE expression is increased in the lipid core of atherosclerotic lesions and at a lesser extent in the fibrous cap, mainly in SMC. The expression of 4-HNE adducts is increased in the adventitia, probably associated with vasa vasorum, which could play a role in the neovascularization of atherosclerotic lesions as reported [31], [32]. 4-HNE-adducts are present in the media, though less marked than in the other two layers. It is significantly age-dependent and mainly associated with SMC, which represent a main source of 4-HNE in this layer.

### Elastin is not modified by HNE-adducts in human aortas

**Fig. 3:**
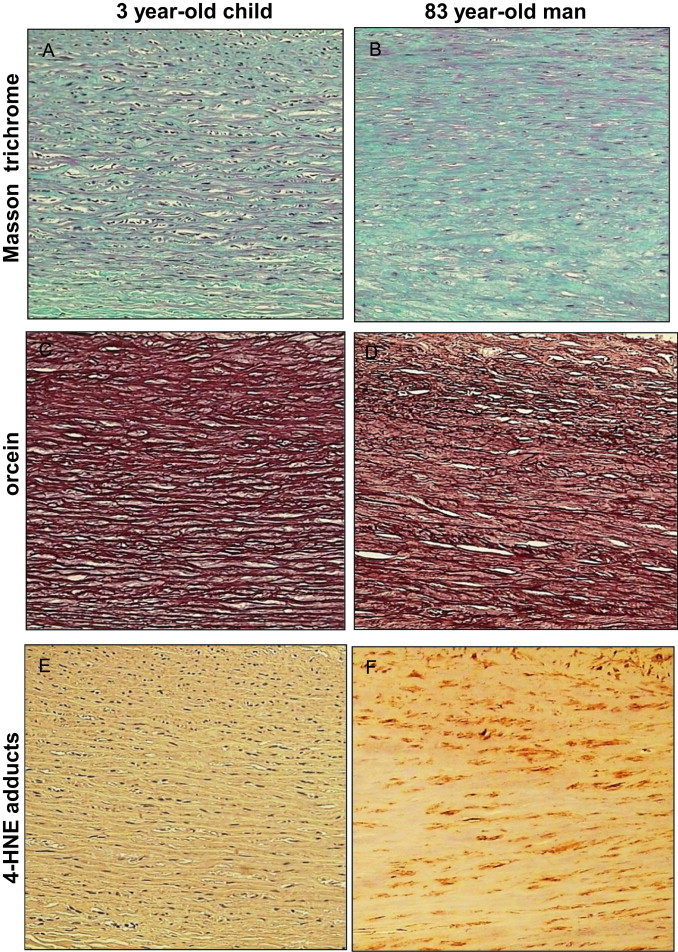
Comparison of HNE-adducts and elastin in young and old aortas. Characterization of aortas from two subjects aged 3 and 83 year-old respectively. Each aorta was characterized by Masson trichrome which stains collagen and elastin (A,B), orcein, which is specific of elastin and elastic tissues (C,D), and 4-HNE adducts (E,F). -Masson trichrome: aorta of the 3 year-old child (left) shows a pattern of normal, parallel elastic, gentle fibers that are light-red stained, while the aorta of 83 year-old man (right) shows thin, fragmented elastic fibers embedded in abundant connective tissue that separates and disrupts parallel order of elastic fibers (Masson’s trichrome, 200×). -Orcein: aorta of the 3 year-old child (left) shows “parallel”, densely arranged elastic fibers, while the aorta of 83 year-old man shows broken, separated and disorganized elastic fibers, without the “parallel” arrangement (orcein 200×).-4-HNE: in the aorta of the 3 year-old child (left) immunohistochemical reaction to 4-HNE shows the absence of 4-HNE in elastic fibers, in SMC and in connective tissue, while in the aorta of 83 year-old man (right) the 4-HNE immunopositivity appeared in SMC, but not in elastic fibers (4-HNE, 200×).

**Fig. 4:**
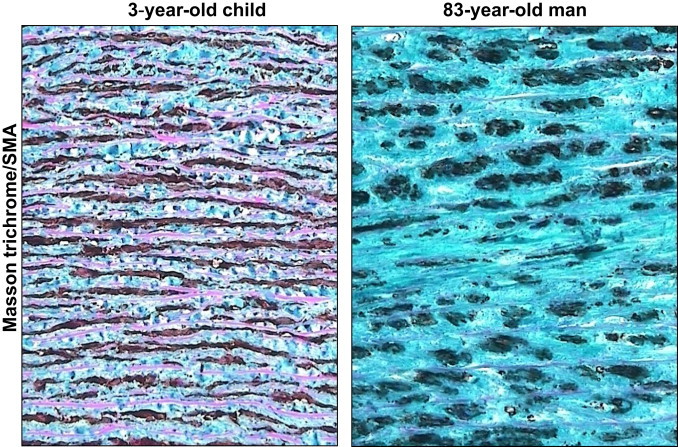
High magnification of Masson trichrome/SMA staining in very young and very old aortic media. Aorta of the 3 year-old child (left) shows a parallel and dense distribution of SMC between elastic fibers. In the aorta of 83 year-old man (right), there is a reduced number of SMC and thick sporadic elastic fibers in an abundant connective tissue (SMA with Masson’s trichrome contrast staining, 400×).

**Fig. 5:**
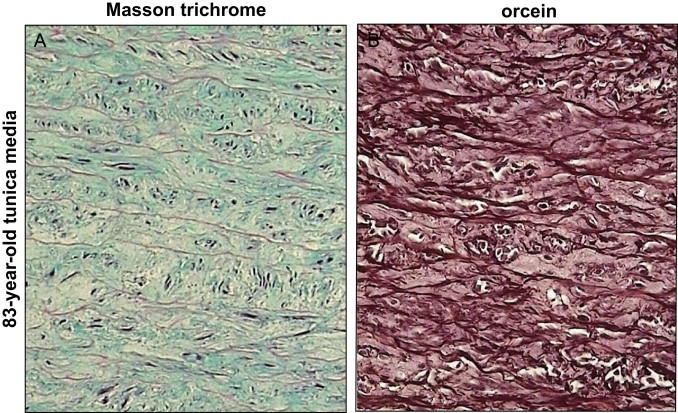
Thin and spaced elastic fibers in old tunica media. Tunica media of the aorta of 83-years old man shows a thin and spaced elastic fibers in the tissue stained by trichrome Masson’s method (left), while in tissue stained by orcein (right) elastic fibers look ticker (400×).

Our data indicate that, as expected, the structure of elastin is deeply modified in an age-related manner. As illustrated in Fig. 3, and at higher magnification in Fig. 4, Fig. 5, orcein and Masson trichrome staining point out an age-related increase of the intralamellar space between elastic fibers in the lamina elastica and the media by comparison with the aorta from a very young child (Fig. 4). This increased interlamellar space is primarily caused by the formation of connective tissue between the elastic lamina, due to the loss of elastic fibers and SMC, as illustrated by the Masson trichrome/SMA staining in the Fig. 4 that points out the strong difference between young and very old media structure.

It should be mentioned that within a same aorta, in particular in elderly persons, various types of histopathological changes occurred simultaneously and were often of various degree, reflecting the chronic character of atherosclerosis. Thickening of elastic fibers appeared as black-thickened aggregates of fibers (Fig. 4, Fig. 5). Such findings are very often related to the findings of the attenuated, spaced and fragmented thin elastic fibers of lamina elastica and tunica media in histological slides stained by Masson’s trichrome technique. The thickened appearance of the orcein-stained elastic fibers probably reflected the modified elastoid substance on the surface of elastic fibers.

**Fig. 6:**
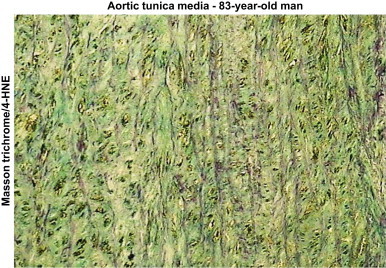
High magnification of Masson trichrome/HNE staining in old tunica media. Aortic media of an 83-year-old man with positive immunochemical reaction to 4-HNE in smooth muscle cells (darker brownish color), but not in elastic fibers and connective tissue (4-HNE with Masson’s trichrome contrast staining, 400×).

4-HNE-adduct expression in the media was present in SMC in the expanded intralamellar area (Fig. 3, Fig. 6). No 4-HNE adducts were detected on elastin fibers and in the connective tissue surrounding the elastic fibers (Fig. 6). The intensity of 4-HNE staining was correlated with aging, as shown in the Fig. 2.

**Fig. 7:**
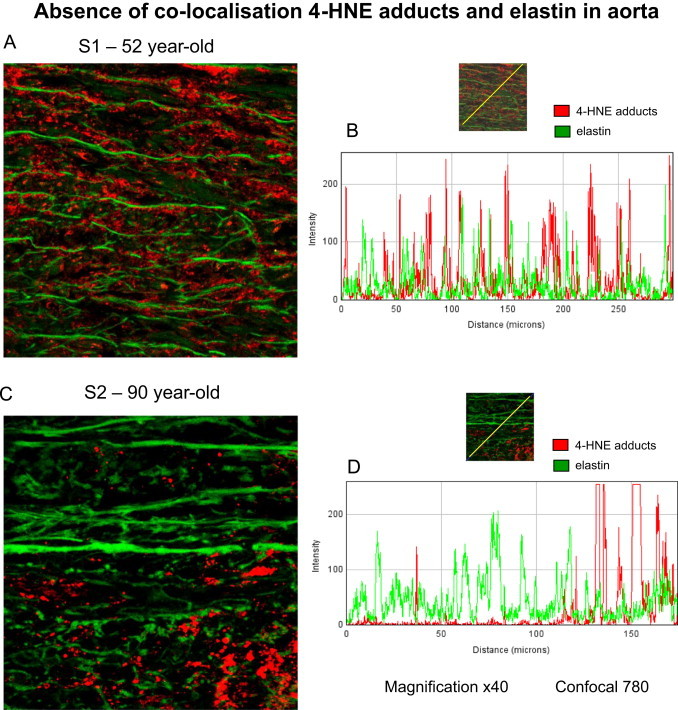
4-HNE adducts and elastin do not colocalize in human aortas. Immunofluorescence and confocal microscopy were done on aortas recovered from two female subjects aged 52 year-old and 90 year-old respectively. Formaldehyde-fixed aorta segments were labeled with anti-4-HNE Michael adduct polyclonal (Calbiochem), and anti-elastin monoclonal antibody (Santa Cruz), and with secondary antibodies Alexa Fluor 488 anti mouse and Alexa Fluor 546 anti-rabbit IgG from Molecular Probes (Invitrogen), as described in the Material/Methods section. The confocal composite image (merge) (× 40 magnification), was analyzed using ImageJ 1.49j software, and confirmed the lack of co-localization between elastin (green peaks) and 4-HNE adducts (red peaks). These data are representative of 4 separate experiments.

Immunofluorescence and confocal studies confirmed the lack of co-localization between 4-HNE-adducts and elastin (Fig. 7). The co-localization of elastin and 4-HNE adducts was studied in the aortas of two patients aged 52 and 90 year old respectively, using an anti Michael-adduct antibody (Calbiochem), which recognizes several 4-HNE epitopes namely histidine-, cysteine- and lysine-4-HNE adducts. Analysis of the results using the Image J129j software, confirmed the accumulation of 4-HNE adducts in the media, and the age-dependent alterations of elastin structure. Moreover, these data confirmed that 4-HNE does not form adducts on aortic elastin, since no or very slight co-localization was observed under confocal microscopy and analysis of the merged pictures.

Altogether, these results emphasize the age-related accumulation of 4-HNE-adducts in human aortas, and indicate that arterial elastin is not a direct target of 4-HNE in the media, in contrast to AGEs [19], [20]. These results do not rule out a role for 4-HNE in elastin alterations and loss occurring in aged arteries.

## Discussion

The aim of this study was to check whether age-related elastin modifications observed in human aortas are correlated with an increased accumulation of HNE-adducts (as a marker of lipid peroxidation that increases in aged human tissues), and whether elastin is modified by 4-HNE in human vessels. Our results indicate that HNE-adducts accumulate in human aorta in an age-related manner, mainly in the intima and the adventitia, and at a lesser extent in the media. 4-HNE-adducts are mainly cellular, and are not detected on elastin, even in elderly, indicating that elastin is not a target of ALEs in the vascular wall. Interestingly, in some patients, the presence of elastic tissue stained black with orcein, but not recognized by other elastin probes, pointed out the accumulation of an elastoid material, comparable to the elastotic material which accumulates in the skin, in the onset of actinic elastosis development.

The first observation is that 4-HNE-adducts are detected in human aortas and their content increases with age, in agreement with the fact that causal links between PUFA levels, 4-HNE adduct formation and longer lifespan have been established and discussed [22]. Importantly, 4-HNE adducts are present in SMC in the aortas of young patients (less than 35 years old, no atherosclerosis development), even in a very young (3 year-old) child. 4-HNE is rapidly generated from PUFA oxidation, under various physiological and pathological situations, independently of aging [33], [34]. A dual role has been reported for 4-HNE, at least *in vitro* on cultured cells, where low intracellular concentrations (0.1–5 µM), could promote ‘hormesis’,, i.e. protection *via* exposure to low stressor concentrations, characterized by increased antioxidant defenses, and survival cellular responses such as cell migration, angiogenesis, proliferation or autophagy [35], [36], while higher concentrations are proinflammatory and proapoptotic [34], [37]. Noteworthy, the post-translational modifications of proteins elicited by 4-HNE, lead first to a gain of function which modulates cell signaling [38], [39], [40]. Thus it can be expected that the presence of 4-HNE in youngers is a marker of transient oxidative stress, which could be considered as a defense mechanism to provoke cell survival, proliferation and compensatory signalings. On the other hand, it is likely that detoxifying enzymes (glutathione S-transferases, aldose reductase, aldehyde dehydrogenases) and the proteasome system [41] are fully efficient at controlling the 4-HNE concentration in youngers, and its removal from the environment once formed, whereas in elderly, these systems are less active and 4-HNE adducts accumulate on proteins, causing dysfunction and apoptosis.

The accumulation of 4-HNE-adducts is very high in the intimal aorta, mainly in older patients with high atherosclerosis grade. These data were expected since oxidized LDL and lipids accumulate in the intima in the early lesions and in the lipid core of advanced atherosclerotic lesions [23], [24]. These data confirm that 4-HNE is a main marker of oxidative stress and LDL oxidation which could contribute to the evolution of the lesions *via* its ability to modify proteins and generate cell dysfunction. 4-HNE expression was also increased in the adventitia of the elderly, probably associated with the vasa vasorum, which are involved in the supplying of nutrients and oxygen to atherosclerotic lesions, and the development of angiogenesis in the atherosclerotic plaque [32], [42]. The recently reported angiogenic effect of 4-HNE suggests a role for this aldehyde in the development of vasa vasorum and microcapillaries in atherosclerotic plaque [35], [43]. Noteworthy, we did not detect any difference in HNE-adduct staining between normotensive and hypertensive patients. However, this does not rule out a role for hypertension in the generation of HNE, because most hypertensive patients in this study were very old and presented high grade of atherosclerosis, which may minimize the implication of hypertension in 4-HNE generation.

More generally, the expression of HNE-adducts in the media was lower than in the intima and adventitia layers, and it was mainly associated with SMC. Importantly, our data show that aortic elastin is not modified by 4-HNE, even in the oldest patients exhibiting evident features of elastin degradation and loss. These data indicate that elastin in the aorta is not a target of 4-HNE, (nor acrolein, data not shown), in contrast to AGEs which were detected on elastin in diabetic patients [19], [20]. These data also differ from the results observed in human skin elastin, showing that elastin and 4-HNE co-localize in actinic elastosis lesions [28]. Several hypothesis could be proposed explaining the fact that 4-HNE, though present, does not modify elastin.

• A first hypothesis could be that histidine epitopes which are the main target for 4-HNE, are present at a very low level on aortic elastin [44], and are probably not available for modification by 4-HNE, in the aortic elastin structure. However, immunofluorescence and confocal studies which confirmed the lack of co-localization between elastin and 4-HNE, were performed using an anti-Michael-adduct antibody, which recognizes histidine, cysteine and lysine epitopes, this indicating that 4-HNE does not bind any of its usual targets on elastin.
• Another possibility is that available lysine epitopes are already modified by AGEs in oldest subjects, since the existence of AGE-adducts has been reported on elastin [19], [20]. This could also explain why acrolein-adducts were not detected, since acrolein-adducts are mainly formed on lysine. However, it should be noted that elastin protein is formed from its precursor tropoelastin, through a massive cross-linking of lysines catalyzed by lysyl oxidase, which generates the mature insoluble and stable elastin [45]. Thus it is likely that lysine epitopes are very poorly available for aldehyde modification in mature elastin in the vessels.

Elastin is a long-life protein, which is not or very poorly renewed, even in case of elastin loss, and particularly in the vessel. Interestingly, we recently reported that 4-HNE inhibits tropoelastin synthesis in fibroblasts, by forming adducts on the EGF receptor, which stimulates EGFR signaling, this counteracting the elastogenic activity of TGF-β [46]. Moreover, 4-HNE could increase the expression of metalloproteases, thereby contributing to elastin degradation [47], [48]. Thus it can be hypothesized that 4-HNE, even if it does not directly modify elastin, contributes to its degradation, and to impair its potential renewal [46].

It is to note that elastin in the skin could be modified by 4-HNE as reported by Tanaka et al. [28] in actinic elastosis lesions in humans. In agreement with these data, our preliminary results indicate that acrolein-adducts are present on elastin in human skin, in contrast with aorta (data not shown). The mechanisms involved in skin elastin modification are not so far identified, nor the structure of modified elastin (intact elastin, fragments, tropoelastin…). AGEs such as Nε-(carboxymethyl)lysines also modify skin elastin, rendering it less digestible for leukocyte elastase, a mechanism probably involved (at least in part), in the accumulation of elastotic material [49]. Preliminary results in our group, indicate that elastin can be *in vitro* modified by acrolein and 4-HNE, which progressively inhibits its degradation by elastase. So, the fact that elastin in aorta is not modified by lipid oxidation products means that aortic and skin elastin do not share exactly the same structure, or that elastin epitopes that could be modified by aldehydes in the skin, are not formed in aortas, or that the modification of elastin in the skin occurs on tropoelastin, since a neosynthesis of elastin could be involved in the accumulation of elastotic material in the onset of photoaging [50]. Noteworthy, the presence of “elastoid” material was observed in the aortas of old subjects, pointing out similarities between skin and vascular elastin aging, though no direct modification of elastin by 4-HNE was observed in this study. Further studies will be necessary for identifying the mechanism of elastin aging in arteries and the targets and role of 4-HNE in this process.
